# Does Clinical Documentation Reflect How Parents and Clinicians Share Decisions About Surgery?

**DOI:** 10.21203/rs.3.rs-7632657/v1

**Published:** 2025-10-06

**Authors:** Angele Kelly, Dalia Mitchell, Anne R. Links, Mary C. Beach, Emily F. Boss, Elaine C. Thompson

**Affiliations:** Johns Hopkins Medicine; The University of Texas Southwestern Medical Center; Johns Hopkins Medicine; Johns Hopkins Medicine; Johns Hopkins Medicine; Johns Hopkins Medicine

**Keywords:** shared decision-making, patient-physician communication, family-centered care, pediatric surgery

## Abstract

**Background:**

While use of shared decision-making (SDM) is widely accepted and applied in practice, it is unknown how core elements of SDM are reflected in clinical documentation. This knowledge gap is uniquely pertinent in the setting of surgery in children, where parents serve as proxy decision-makers.

**Methods:**

We analyzed transcribed audio-recorded outpatient encounters and corresponding medical documentation for pediatric patients undergoing evaluation for elective surgery. Visit transcripts and medical record documentation of the corresponding clinical encounter were coded for two core elements of shared decision-making: parental concerns and preferences. We assessed the frequency, content, and timing of these elements expressed in verbal communication and in clinical documentation.

**Results:**

Of 109 visits, concerns and preferences were discussed in nearly half of encounters (Concerns n = 46, 42%; Preferences n = 49, 45%). Most verbally-expressed concerns (e.g., “we’re worried about her breathing”; “…she sleeps with me. I’m constantly shaking her. She scares me a lot”) focused on symptoms and were raised throughout the encounter. Preferences (e.g., “I would just rather take them out”; “I’d definitely rather go the conservative route”) centered on treatment decisions and were typically expressed later in the visit, after the physical exam. Of the total unique stated concerns (n = 74) and preferences (n = 62), about half were recorded in the medical record (Concerns n = 34, 46%; Preferences n = 28, 49%).

**Conclusions:**

These findings show that parent preferences and concerns that are discussed during pediatric surgical encounters are not routinely reflected in clinical documentation. Improving the consistency with which family perspectives are documented may enhance transparency, support communication across the health continuum, and contribute to high quality patient- and family-centered care.

## Background

Shared Decision-Making (SDM) is a collaborative process where clinicians, patients and families collectively determine a treatment plan based on best available evidence and patient values [1, 2]. Engaging the patient and family in decisions promotes higher quality treatment and healthcare delivery, and potentially reduces unwarranted variation in care [3, 4]. Shared decision-making prioritizes inclusion of patient and family preferences and concerns to inform decision for treatment [5]. Shared decision-making has been explored across a variety of medical fields but has unique relevance in the context of pediatric elective surgery, where treatment decisions involve parents as proxies and often clinical equipoise [1, 6, 7]. Despite an established recognition of the importance of shared decision-making, implementation in clinical practice is difficult to measure [8].

Little is known about how core elements of shared decision-making, especially patient or parent preferences or concerns, are documented by clinicians during pediatric surgical consultations. Understanding how these elements are reflected in clinical documentation may promote safe, patient- and family-centered care and allow for facile measurement of the SDM process and correlation with patient outcomes [9]. Documentation of shared decision-making is particularly relevant given the recent emphasis on transparency of patient medical records permitting open access to the medical record [10].

In this study, we examine whether and how core elements of shared decision-making in clinical encounters (parent preferences and concerns) are documented in the medical record. We analyzed transcribed audio-recorded verbal communication occurring during surgical encounters alongside clinical documentation of the encounters to determine the extent to which these elements exist and correspond to one another.

## Methods

### Participants

Data analyzed in this study is part of a larger investigation of shared decision-making and patient outcomes in pediatric surgical care [5, 11]. All procedures were approved by the Johns Hopkins School of Medicine Institutional Review Board (IRB54662) and were performed in accordance with the Declaration of Helsinki. Participants included clinicians and parents/guardians of patients presenting to one of three outpatient surgery clinics at Johns Hopkins Medicine: pediatric otolaryngology, urology, or general surgery. Informed written consent was obtained from all participants, and patients were enrolled over a 3-year period. Clinical encounters were audio-recorded and transcribed verbatim. Parents and clinicians completed demographic questionnaires.

### Experimental design

Mixed-methods analysis was used to examine elements of shared decision-making within verbal encounters and clinical documentation. Qualitative analysis involved coding dialogue between clinicians and parents during the visit (transcribed encounter) according to previously defined SDM themes to indicate presence of each element (parental preferences and concerns) in the transcript. The coded transcript communication was then compared to clinical documentation (electronic health record) to assess if and when it was present. Quantitative analysis involved descriptive statistics of parental preferences and concerns within the two types of communication (verbal and written). Microsoft Excel 2021 and SPSS 29 [12] were used.

### Data analysis

Both clinical encounter transcripts and corresponding medical documentation were reviewed for two core elements of shared decision-making: expression of parental preferences and parental concerns. These themes were selected prior to analysis based on previous research evaluating central elements of shared decision-making in elective pediatric surgery [1, 5, 13] and based on initial comparative review of transcripts and clinical documentation. We chose to exclusively focus on these elements because in our preliminary analysis, we noted that additional features, such as documentation of treatment alternatives, risks, and benefits, were predominantly included using electronic templates ( “dot phrases”, or smart text).

Parental preferences were defined as any preference or choice clearly expressed during the encounter. Parental concerns were defined as any instance in which a parent expressed worry, apprehension, or fear during the encounter, typically related to symptoms, risks of surgeries, or similiar. Preferences and concerns were further categorized into topics (symptoms, treatment decision, risks of anesthesia, risks of surgery, surgical plan/logistics, treatment timeline, or other) and timing (occurring early or late in the encounter). “Early” and “late” were defined as occurring prior to or after the physical exam, respectively. Medical documentation of preferences/concerns reported as part of an electronic template was excluded.

One coder (AK) independently evaluated all transcripts and clinical documentation to abstract the identified elements. A subset of these transcripts (35%, n = 38) were double-coded by a second researcher (ET) and assessed for interrater agreement [14]. Full resolution was reached between both coders (AK and ET). The entire research team met routinely to review coding and resolve differences.

## Results

### Demographics/participant info

Participants included 16 clinicians who engaged in a cumulative 109 clinical visits to evaluate children for elective surgery including tonsillectomy (n = 96; 88%), inguinal or umbilical hernia repair (n = 9, 8%), or circumcision (n = 4, 4%). Nine (56%) clinicians were male and 12 (75%) were White, one was Asian, one was Hispanic, and two were of multiple races/ethnicities. Clinician ages ranged 31–60 (M = 40 +/− 7.45).

Parent/guardian participants were mostly female (n = 95, 87%) with ages ranging from 23–66 (M = 35 +/− 7.46). Fifty-three parents were White (48%), forty were Black (37%), one was Hispanic, one Asian, and two were multiples races. There was a balanced distribution across insurance types (private: n = 59, 54%), income status ($20,000-$49,999 n = 28, 26%), and education level (2-year degree or some college n = 33, 30%;) (Table 1).

### Agreement

Final coding represents full consensus between coders (AK and ET). The interrater reliability for the raters was substantial to perfect across all variables [Transcript preferences Kappa = 0.797 (p < 0.001), 95% CI (0.613, 0.981); Transcript concerns Kappa = 0.851 (p < 0.001), 95% CI (0.690, 1.012); Medical record preferences Kappa = 0.918 (p < .0.001), 95% CI (0.761, 1.075); Medical record concerns Kappa = 0.632 (p = 0.004), 95% CI (0.256, 1.008)].

### Parental concerns and preferences

Parental concerns were expressed in 42% (n = 46) of encounters. There were 74 unique concerns stated across 46 visits. Of the 74 concerns, 34 (46%) had corresponding documentation in the medical record. Of these 34 instances, 33 (97%) matched the verbalized concerns (concordant), and one did not (discordant). In the discordant example, the patient expressed concern about the child falling behind in school because of absences related to illness, and the clinician reported “doing well in school” in the EMR. More than half 54% (n = 40) of visits did not have corresponding data in the medical record. Concern categories were largely related to symptoms (n = 42, 57%), followed by surgery risk (n = 7, 9%) and treatment decisions (n = 6, 8%). A little over half of the concerns occurred were expressed early in the encounter (n = 41, 55%) during the history taking portion. Please see Tables 2 and 3 for further details.

Parental preferences were expressed in 45% (n = 49) of encounters. There were 62 unique preferences expressed across these 49 visits. Of the 62 preferences, 28 had corresponding information documented in the medical record (45%); of these 28 instances, all were concordant. Corresponding documentation was absent in more than half of 55% (n = 34) of the visits. The most common preference category was treatment decision (n = 48, 77%), followed by surgical planning/logistics (n = 10, 16%) and timing of treatment (n = 3, 5%). Almost all unique preferences were expressed late in the encounter after physical exam (n = 59, 95%). (Tables 2 and 3).

## Discussion

Use of shared decision-making during clinical encounters has been previously explored through analyses using descriptive or qualitative coding frameworks as well as quantitative communication coding scales [5, 15, 16]. To our knowledge, this is the first study to compare how parent-expressed preferences and concerns during pediatric surgical consultations correspond with clinical documentation. Nearly half of encounters included verbal expressions of concerns or preferences, yet these elements were reflected in less than half of the documentation. Notably, when documentation did occur, it closely aligned with what was said in the encounter. Together these findings suggest that shared decision-making is occurring in real time but is not always documented.

Characterizing concerns and preferences is an important step for understanding patient/family values that guide treatment decisions. In the setting of pediatric surgical care, patients and families are faced with choices that require understanding and weighing of operative risks and treatment alternatives. Guidance from surgeons may help families to make informed health care decisions that align with their priorities, improving quality of decisions and reducing parental decisional conflict [17]. Prior work has explored concerns and preferences as elements of shared decision-making in pediatric surgical care through qualitative analysis using in-depth interviews [13, 18], analysis of internet-based parent-reported perspectives [19], and thematic analysis of visit transcripts [5]. Our research adds to this literature by incorporating how parent concerns and preferences are documented in the medical record, which has not been explored in this context to date.

Documentation carries increasing importance in today’s healthcare landscape. Under the 21st Century Cures Act, families are entitled to full access of their child’s medical records [20]. Beyond promoting transparency, documentation also serves clinical and legal functions. For example, some insurers require that shared decision-making be documented to authorize treatment, such as for implantable cardioverter defibrillators (ICDs) [21]. Clinical documentation can also shape downstream care, as the content of medical notes has been shown to impact clinical outcomes [22]. Although shared decision-making is not regulated for the majority of clinical settings, including pediatric surgical consultations, sparse documentation of parent perspectives may give the false impression that shared decision-making did not occur. Further, as documentation practices evolve, especially with the integration of ambient artificial intelligence scribes, attention to how shared decision-making is captured and measured will be important for standardizing documentation practices.

From the family perspective, access to clinical notes improves patient/family knowledge of diagnosis and treatment plans [23] and fosters trust between patient families and clinicians [24, 25]. Documentation can also improve interdisciplinary communication by clarifying what matters most to families, including cultural values, logistical barriers, or fears about treatment [9]. Our finding that more than half of notes in this study did not reflect verbally-expressed concerns or preferences suggests there may be opportunity to better communicate essential family context to other members of the care team through documentation. This has implications for clarifying how social determinants of health impact care decisions [9].

Our study has limitations. First, identifying concerns and preferences in verbal communication is inherently subjective, and some instances may have been missed despite careful review. Next, while we excluded templated content within clinical documentation, we recognize that clinicians may sometimes use such phrases intentionally. Additionally, we did not analyze documentation patterns by race, education, or gender, which are important areas for future research given existing disparities in patient-clinician communication [26]. Because our project aimed to simply identify preferences and concerns, we did not perform analyses to account for clinician clustering. Finally, we did not evaluate whether the patient or clinician initiated the topic of concerns/preferences, which may also implicitly or explicitly influence clinician recall or intent in documentation. Despite these limitations, our findings highlight descrepancies between what is communicated verbally during clinical encounters and what is documented in medical records, revealing opportunities to enhance transparency, continuity, and family-centeredness in pediatric surgical care.

## Conclusions

This study demonstrates that parental concerns and preferences are frequently expressed during pediatric surgical consultations but are not consistently documented in the medical record. Improving documentation of family values and treatment priorities may enhance transparency, support continuity of care, and facilitate communication across providers. Better alignment of physician-patient interpersonal communication and medical documentation may also serve as a measurable indicator of patient- and family-centered care quality in pediatric surgical settings.

## Figures and Tables

**Table 1.** Demographics of patients and clinicians.

	Participants mean +/− SD or n (%)	
	
Demographic Variable	Parents (n = 109)	Clinician (n = 16)
	
Age, y	34.54 +/− 7.46	
Race/ethnicity		
White	53 (48)	12 (75)
Black	40 (37)	0 (0)
Hispanic	8 (7)	1 (6)
Asian	4 (4)	1 (6)
Other	4 (4)	2 (13)
Gender		
Male	13 (12)	9 (56)
Female	95 (87)	7 (44)
Unlisted	1 (1)	
Insurance type		NA
Private	59 (54)	
Public	50 (46)	
Annual Household Income		NA
<$20,000	22 (20)	
$20,000–$49,999	28 (26)	
$50,000–$79,000	16 (14)	
$80,000–$100,000	12 (11)	
>$100,000	27 (25)	
Prefer not to say	4 (4)	
Education		NA
Less than high school degree	6 (6)	
High school degree	21 (19)	
Some college	33 (30)	
Bachelor’s degree	25 (23)	
Graduate-professional degree	23 (21)	

**Table 2.** Characteristics of expression and documentation: frequency, category, and timing of concerns and preferences

Documentation characteristics	n (%)

Visits coded (unique patient encounters)	109 (100)

Visits with at least one concern	46 (42)
Total unique concerns	74
Mean concerns per visit (+/− SD)	1.61 (+/− 0.95)
Range	0–5
Corresponding documentation	34 (46)
No corresponding documentation	40 (54)

Unique concerns by category	
Symptoms	42 (57)
Risk of surgery	7 (9)
Management decision	6 (8)
Risks of anesthesia	5 (7)
Other (surgical planning, risk of sleep apnea, etc.)	14 (19)

Timing of unique concerns	
Early	41 (55)
Late	33 (45)

Visits with at least one preference	49 (45)
Total unique preferences	62
Mean preferences per visit (+/− SD)	1.27 (+/− 0.66)
Range	0–5
Corresponding documentation	28 (45)
No corresponding documentation	34 (55)

Unique preferences by category	
Management decision	48 (77)
Surgical plan/logistics	10 (16)
Treatment timeline	3 (5)
Other	1 (2)

Timing of unique preferences	
Early	3 (5)
Late	59 (95)

**Table 3:** Examples of concerns and preferences expressed in verbal communication and corresponding clinical documentation.

Concern or preference		Dialogue (from encounter)	Corresponding clinical documentation	
	Category		Present/Absent	Example
Concern	Symptom	“Well, I shake her and that’s why we had the sleep study done... because she sleeps with me. I’m constantly shaking her. She scares me a lot.”	Present	“Witnessed apneas.”
Concern	Symptom	“Well, the reason I’m kind of concerned over the, like, sleep issues is the reports of a bit of lethargy. So they’re saying he’s not being super focused that instructions are having to be, um, repeated. they are... um, wanting to pursue ... testing ...which falls in that ADHD thing, And with, I’m tending to lean more going completely, like neuro-psych eval”	Present	“Symptoms include fatigue and lethargy during the day as well as snoring and restless sleep. ADD workup being considered”
Concern	Management decision	“I’m a little nervous about doing the sleep study.”	Absent	NA
Concern	Anesthesia risk	“Um, and I’ve never wanted him to have surgery again with the anesthesia... ‘cause I’ve just paranoid about he would recover.”	Absent	N/A
Preference	Treatment decision	“I checked online for a lot of knowledge already, and uh so I- mm opinion is to do a surgery as soon as possible actually, specifically came to this doctor because they read that the intracapsular procedure is less painful for kids and they would prefer it.”	Present	“Patient’s father would like to proceed with surgery.”
Preference	Treatment decision	“I mean if it’s not an issue then um, if it’s not broke don’t fix it.”	Present	“Elected observation for now.”
Preference	Treatment decision	“I think we should go ahead with the sleep study first... Before we go with the surgery because I think that would be like our last resort”	Present	“After review of the options, parent elected for sleep study.”
Preference	Treatment decision	“Um, well, if we’re gonna try the spray, that’s, you know, I prefer that first.”	Absent	N/A

* N/A represents instances where preferences or concerns were discussed in the encounter but were not documented in the electronic medical record.

## Data Availability

The dataset supporting the conclusions of this article is not posted publicly in order to respect individual privacy, but is available upon email request to lead author.

## Abbreviations

SDM: Shared decision making
SPSS: Statistical Package for Social Sciences
n: number

## References

[1] Boss EF, Mehta N, Nagarajan N, Links A, Benke JR, Berger Z, Shared Decision Making and Choice for Elective Surgical Care: A Systematic Review. Otolaryngol Neck Surg. 2016;154:405–20. 10.1177/0194599815620558. PMC4857133 26645531

[2] Elwyn G, Durand M-A. Mastering Shared Decision Making: The When, Why and How. 2017.

[3] Frosch DL, Moulton BW, Wexler RM, Holmes-Rovner M, Volk RJ, Levin CA. Shared decision making in the United States: policy and implementation activity on multiple fronts. Z Evidenz Fortbild Qual Im Gesundheitswesen. 2011;105:305–12. 10.1016/j.zefq.2011.04.004. 21620326

[4] Oshima Lee E, Emanuel EJ. Shared decision making to improve care and reduce costs. N Engl J Med. 2013;368:6–8. 10.1056/NEJMp1209500. 23281971

[5] Callon W, Beach MC, Links AR, Wasserman C, Boss EF. An expanded framework to define and measure shared decision-making in dialogue: A ‘top-down’ and ‘bottom-up’ approach. Patient Educ Couns. 2018;101:1368–77. 10.1016/j.pec.2018.03.014. 29550295 PMC6475620

[6] Carlisle EM, Klipowicz CJ, Shinkunas LA, Scherer AM, Kaldjian LC. Discrepancies in decision making preferences between parents and surgeons in pediatric surgery. BMC Med Inform Decis Mak. 2021;21:42. 10.1186/s12911-021-01414-z. 33541347 PMC7863410

[7] Carlisle EM, Shinkunas LA, Ruba E, Klipowicz CJ, Lieberman MT, Hoffman RM, A valued voice: A qualitative analysis of parental decision-making preferences in emergent paediatric surgery. Health Expect Int J Public Particip Health Care Health Policy. 2023;26:531–41. 10.1111/hex.13686. PMC9854285 36482826

[8] Ikeda AK, Hong P, Ishman SL, Joe SA, Randolph GW, Shin JJ. Evidence-Based Medicine in Otolaryngology Part 7: Introduction to Shared Decision Making. Otolaryngol--Head Neck Surg Off J Am Acad Otolaryngol-Head Neck Surg. 2018;158:586–93. 10.1177/0194599818756814. 29406794

[9] Thompson EC, Boss EF. Improving Care Quality Through Documented Shared Decisions. Qual Manag Health Care. 2025;34:266–7. 10.1097/QMH.0000000000000544. 40643225 PMC12631752

[10] Tapuria A, Porat T, Kalra D, Dsouza G, Xiaohui S, Curcin V. Impact of patient access to their electronic health record: systematic review. Inform Health Soc Care. 2021;46:192–204. 10.1080/17538157.2021.1879810. 33840342

[11] Leu GR, Links AR, Tunkel DE, Walsh JM, Ryan MA, DiCarlo H, Understanding Bias in Surgery: Perceived Cultural Similarity Between Surgeons and Patient Families. Otolaryngol Neck Surg. 2021;165:282–9. 10.1177/0194599820982639. 33430701

[12] SPSS Statistics.

[13] Boss EF, Links AR, Saxton R, Cheng TL, Beach MC. Parent Experience of Care and Decision Making for Children Who Snore. JAMA Otolaryngol Neck Surg. 2017;143:218. 10.1001/jamaoto.2016.2400. PMC5798224 27560831

[14] McHugh ML. Interrater reliability: the kappa statistic. Biochem Medica. 2012;22:276–82. PMC3900052 23092060

[15] Evong Y, Chorney J, Ungar G, Hong P. Perceptions and observations of shared decision making during pediatric otolaryngology surgical consultations. J Otolaryngol - Head Neck Surg J Oto-Rhino-Laryngol Chir Cervico-Faciale. 2019;48:28. 10.1186/s40463-019-0351-x. PMC6580583 31208462

[16] Nicolai J, Moshagen M, Eich W, Bieber C. The OPTION scale for the assessment of shared decision making (SDM): methodological issues. Z Evidenz Fortbild Qual Im Gesundheitswesen. 2012;106:264–71. 10.1016/j.zefq.2012.03.002. 22749073

[17] Boland L, Kryworuchko J, Saarimaki A, Lawson ML. Parental decision making involvement and decisional conflict: a descriptive study. BMC Pediatr. 2017;17:146. 10.1186/s12887-017-0899-4. 28610580 PMC5470309

[18] Boss EF, Links AR, Saxton R, Cheng TL, Beach MC. Physician Perspectives on Decision Making for Treatment of Pediatric Sleep-Disordered Breathing. Clin Pediatr (Phila). 2017;56:993–1000. 10.1177/0009922817702939. 28429620 PMC6460467

[19] Hairston TK, Links AR, Harris V, Tunkel DE, Walsh J, Beach MC, Evaluation of Parental Perspectives and Concerns About Pediatric Tonsillectomy in Social Media. JAMA Otolaryngol--Head Neck Surg. 2019;145:45–52. 10.1001/jamaoto.2018.2917. 30452510 PMC6439813

[20] st Century Cures Act: Interoperability, Information Blocking, and the ONC Health IT Certification Program; Final Rule. 2020.

[21] Decision Memo for Implantable Cardioverter Defibrillators (CAG-00157R4). Centers for Medicare & Medicaid Services; 2018.

[22] Goddu A, O’Conor KJ, Lanzkron S, Saheed MO, Saha S, Peek ME, Do Words Matter? Stigmatizing Language and the Transmission of Bias in the Medical Record. J Gen Intern Med. 2018;33:685–91. 10.1007/s11606-017-4289-2. 29374357 PMC5910343

[23] D’Costa SN, Kuhn IL, Fritz Z. A systematic review of patient access to medical records in the acute setting: practicalities, perspectives and ethical consequences. BMC Med Ethics. 2020;21:18. 10.1186/s12910-020-0459-6. 32122332 PMC7053049

[24] Crucefix AL, Fleming APL, Lebus CS, Slowther A-M, Fritz Z. Sharing a written medical summary with patients on the post-admission ward round: A qualitative study of clinician and patient experience. J Eval Clin Pract. 2021;27:1235–42. 10.1111/jep.13574. 33960593

[25] Kelly MM, Hoonakker PLT, Nacht CL, Smith CA, Dean SM, Sklansky DJ, Parent Perspectives on Sharing Pediatric Hospitalization Clinical Notes. Pediatrics. 2023;151:e2022057756. 10.1542/peds.2022-057756. 36450655 PMC9998186

[26] Harris VC, Links AR, Walsh J, Schoo DP, Lee AH, Tunkel DE, A Systematic Review of Race/Ethnicity and Parental Treatment Decision-Making. Clin Pediatr (Phila). 2018;57:1453–64. 10.1177/0009922818788307. 30014706 PMC6460468

